# Vascular biosafety of commercial hydroxyapatite particles: discrepancy between blood compatibility assays and endothelial cell behavior

**DOI:** 10.1186/s12951-018-0357-y

**Published:** 2018-03-22

**Authors:** Catarina Santos, Suzy Turiel, Pedro Sousa Gomes, Elísio Costa, Alice Santos-Silva, Paulo Quadros, José Duarte, Sílvia Battistuzzo, Maria Helena Fernandes

**Affiliations:** 1 EST Setúbal, DEM, Instituto Politécnico de Setúbal, Campus IPS, 2914-508 Setúbal, Portugal; 2 CQE, Instituto Superior Técnico, Universidade de Lisboa, Av. Rovisco Pais, 1049-001 Lisbon, Portugal; 3 Faculdade de Medicina Dentária, U. Porto, Rua Dr. Manuel Pereira da Silva, 4200-393 Porto, Portugal; 4 REQUIMTE/LAQV – U. Porto, Porto, Portugal; 5 UCIBIO/REQUIMTE, Departamento de Ciências Biológicas, Laboratório de Bioquímica, Faculdade de Farmácia, U. Porto (FFUP), Porto, Portugal; 6 FLUIDINOVA, S.A, Moreira da Maia, Portugal; 7 CIAFEL, Faculdade de Desporto, Universidade do Porto, Porto, Portugal; 8 Laboratório de Biologia Molecular e Genômica, Centro de Biociências, Universidade Federal do Rio Grande do Norte (UFRN), Campus Universitário s/n, Lagoa Nova, Natal, RN 59072-970 Brazil

**Keywords:** Vascular compatibility, Hydroxyapatite particles, Blood compatibility, Endothelial cell behaviour

## Abstract

**Background:**

Vascular homeostasis is ensured by a dynamic interplay involving the endothelium, the platelets and the coagulation system. Thus, the vascular safety of particulate materials must address this integrated system, an approach that has been largely neglected. This work analysed the effects of commercial hydroxyapatite (HA) particles in blood compatibility and in endothelial cell behavior, due to their clinical relevance and scarcity of data on their vascular biosafety.

**Results:**

Particles with similar chemical composition and distinct size and morphology were tested, i.e. rod-like, nano dimensions and low aspect ratio (HAp1) and needle-shape with wider size and aspect ratio (HAp2). HAp1 and HAp2, at 1 to 10 mg/mL, did not affect haemolysis, platelet adhesion, aggregation and activation, or the coagulation system (intrinsic and extrinsic pathways), although HAp2 exhibited a slight thrombogenic potential at 10 mg/mL. Notwithstanding, significantly lower levels presented dose-dependent toxicity on endothelial cells’ behavior. HAp1 and HAp2 decreased cell viability at levels ≥ 250 and ≥ 50 μg/mL, respectively. At 10 and 50 μg/mL, HAp1 did not interfere with the F-actin cytoskeleton, apoptotic index, cell cycle progression, expression of vWF, VECad and CD31, and the ability to form a network of tubular-like structures. Comparatively, HAp2 caused dose-dependent toxic effects in these parameters in the same concentration range.

**Conclusion:**

The most relevant observation is the great discrepancy of HA particles’ levels that interfere with the routine blood compatibility assays and the endothelial cell behavior. Further, this difference was also found to be dependent on the particles’ size, morphology and aspect ratio, emphasizing the need of a complementary biological characterization, taking into consideration the endothelial cells’ functionality, to establish the vascular safety of particulate HA.

## Background

Synthesised hydroxyapatite (HA), due to its similarity to the inorganic phase of bone and teeth, has various biomedical applications. As such, it is widely used as bulk and particulate formulations in a variety of bone related applications including repair/regeneration, augmentation and coating materials, alone or as composite/hybrid materials, and more recently in agents for enamel remineralization and toothpastes [1]. The application of HA, however, extends beyond the scope of the hard tissues. HA has the ability to reversibly adsorb a variety of proteins, drugs, other bioactive molecules and particles, finding a potential wide range of applications from drug and gene delivery and targeting therapies, to medical diagnosis [2]. When produced at the nanoscale, the high surface-to-volume ratio and surface reactivity offer enhanced reaction sites and biomimetic interaction, greatly improving its performance and widening its therapeutic and biomedical potential [2].

Both in local applications within bone or teeth (following degradation and diffusion events from implanted materials, or the repetitive application within the oral environment with toothpaste’s use), or in those aiming systemic strategies, HA particles appear in the vascular compartment [3]. Here, particles presenting a wide range of features regarding size, morphology and surface properties contact and interact with the complex cellular and biochemical blood environment [3].

In the vascular compartment, the concerted interaction of endothelium, platelets and coagulation factors ensures the maintenance of vascular and tissue homeostasis. The endothelium, a monolayer of cells lining the blood vessels, is a dynamic interface between blood and non-vascular tissues, creating a barrier to prevent blood components from contacting the sub-endothelial extracellular matrix [4]. Additionally, it has a key role in the modulation of the vascular tone by expressing and secreting vasoactive molecules assisting in the maintenance of an appropriate blood flow and in preventing intravascular platelet activation and blood coagulation [4]. In this integrated system, platelets survey the vascular environment for the integrity of the endothelium [5]. Damage to this continuous cell monolayer exposes the sub-endothelial tissues, which triggers local platelet recruitment, adhesion, activation and aggregation [6]. The on-going activation of the coagulation system leads to the formation of a fibrin network that stabilizes the platelet aggregates, forming a clot in the injured area [6].

In this context, the presence of particulate HA in the vascular environment might affect this dynamic homeostasis, by interacting with endothelial cells, platelets and coagulation factors [7–9]. Thus, the assessment of HA particles in this setting is a requirement for its safety profile, for either bulk or particulate formulations. In spite of that, biocompatibility of HA has been analysed especially addressing the bone tissue [1] and information on its interaction with the vascular compartment is scarce and loose. The few studies are mainly concerned with isolated effects on the platelets and/or the coagulation system [10–13]. Others address only the interaction with endothelial cells and, most of them, in a perspective of angiogenesis and bone regeneration [14–16].

This work proposes a complementary approach to address the vascular effects of HA particles, by analysing the interaction with endothelial cells, platelet adhesion and activation, and coagulation system. The working hypothesis is that the dose-effects of HA particles in these three components might differ significantly, thus a concerted vision is needed for adequate establishment of the vascular safety profile. Two commercially available HA particles, with similar chemical composition but distinct size, morphology and aspect ratio, were used to test this hypothesis.

## Methods

### Hydroxyapatite particles

Commercial HA particles, rod-like (nanoXIM•HAp102^®^, Fluidinova, S. A.) and needle-like (Plasma Biotal Ltd.) were used, and designated respectively as HAp1 and HAp2.

The size and morphology of HA particles were evaluated by transmission electron microscopy (TEM; Hitachi 8100 operating at 200 kV, with ThermoNoran light elements EDS detector). Selective area electron diffraction (SAED) patterns were obtained for both samples. The particles were dispersed in absolute ethanol and deposited onto formvar-coated copper grids that were subsequently air-dried for TEM observation. The size (length and width) of both HA particles were measured using ITEM software. To obtain the particle size distribution 100 HAp nanoparticles were analysed.

The specific surface area was determined by N_2_ adsorption using an Accelerated Surface Area system 2020 from Micromeritics, with 15 s equilibration time. The samples were outgassed for 120 min at 120 °C.

The particles composition was provided by microRaman spectroscopy using a Horiba LabRAM HR800 Evolution (Jobin–Yvon, France) spectrometer. For both samples a solid-state laser operating at 532 nm with a power of 6mW reaching the samples was used. A 50× objective lens (numerical aperture of 0.75) focused the laser beam to a 0.87 μm spot on the samples surface. The spectra were obtained with an acquisition time of 10 s and 10 accumulations. The system was calibrated to better than 1 cm^−1^ before measurements, using a silicon sample. A spectrograph with a 600 lines/mm grating was used to provide a spectral resolution of 2 cm^−1^, and the confocal pinhole size was set at 200 μm.

The crystalline phases of the HA particles were identified by power X-ray diffraction (XRD) analysis using a PANalytical’s X’PerPRO with a Cu-K*α* radiation (k = 1.5418 Å), operated at 45 kV, 40 mA, using a linear X’Celerator detector. The diffraction patterns were recorded at room temperature over a 2*θ* range of 15°–65°. DRX analyses were performed in grazing geometry (GIXRD).

Homogeneous dispersion of the particles in the medium was assured by vortex mix prior to biological testing.

### In vitro blood compatibility assays

Blood compatibility was evaluated for haemolysis, platelet aggregation and activation, and coagulation system. Standard guidelines and widely used methodologies were followed [17–20]. Blood was collected by venipuncture from three healthy non-smoking adult volunteers who were free from any medication for at least 2 weeks. Blood collection and preparation were performed according the ISTH and BCSH Guidelines [3, 17, 19]. Preliminary experiments showed that HA particles did not affect the tested blood parameters at concentration up to 1 mg/mL. Thus, higher levels were used (up to 10 mg/mL) to disclose detectable alterations for, at least, one of the particles.

#### Preparation of platelet-rich plasma (PRP) and platelet-poor plasma (PPP)

For the preparation of PRP and PPP, fifteen millilitres of blood were collected into sodium citrate tubes (S-monovette^®^ 5 mL 9 NC, Sarstedt AG & Co, Nümbrecht, Germany). The first 3 mL of drawn blood were discarded to avoid contamination by thromboplastin released by needle puncture. For PRP, 5 mL of whole blood were centrifuged at 250 g for 15 min and the platelet-rich supernatant was removed. Platelet concentration on PRP was determined using an automated cell blood counter (ABX Micros ES 60, Horiba, Ltd) prior to incubation with samples. In order to obtain PPP, 5 mL citrate tubes were spun at 2000 g for 15 min and the supernatant was collected to a simple tube until further processing.

#### Haemolysis

Blood collected in tubes containing EDTA was immediately centrifuged (405 g, 10 min), and plasma and buffy coat were carefully removed. Erythrocytes were washed with phosphate-buffered saline (PBS, 4 °C) and re-suspended in PBS to obtain a red blood cell (RBC) suspension at 10% (v/v) haematocrit. HAp1 and HAp2 were tested at 1 and 10 mg/mL by incubation with the erythrocyte suspension (37 °C, 3 h), under gentle shaking; incubation of the erythrocyte suspension in PBS was used as control. Haemolysis was quantified with UV/Vis spectroscopy by measuring free plasma haemoglobin (λ = 540 nm) from erythrocytes’ destruction. Results are presented as haemolysis percentage.

#### Platelet morphology and aggregation

For the evaluation of platelet morphology, PRP was incubated with HAp1 and HAp2 (10 mg/mL, 2 h, 37 °C) over standard cell culture coverslips (TCPs) placed in the wells of a 24-well plate (13 mm diameter), using 200 μL/well. PRP incubated on polypropylene and glass surfaces of similar dimensions were used as negative and positive controls, respectively. Samples were washed with PBS and the adherent cells were fixed (3.7% paraformaldehyde, 15 min), dehydrated with a graded (70, 80, 90, and 100%) ethanol series, critical point dried, coated with an Au/Pd thin film (SPI Module Sputter Coater equipment) and observed under a high resolution environmental SEM (Quanta 400 FEG ESEM).

Platelet aggregation was assayed by light aggregometry using a lumi-aggregometer (Chrono-Log, Manchester, UK). Briefly, PRP samples (200 μL) were incubated in the presence of nanoparticles (HAp1 or HAp2—10 mg/mL), and their effects were recorded for 15 min. Collagen (5 μg/mL), a known inductor of platelet aggregation was used as a positive control and PRP alone was considered the negative control.

#### Platelet expression of CD42a/GP9 and CD62P

HAp1 and HAp2 (1 and 10 mg/mL), polypropylene beads (negative control) and glass beads (positive control, Sigma Aldrich, 18406-500G, St. Louis) were incubated in Eppendorf tubes in triplicate with PRP (100 μL/tube; 2 h, 37 °C) under agitation. After incubation, platelets suspension was diluted five-fold in physiologic saline, in order to minimize the presence of aggregates. Platelets were stained with anti-CD42a/GP9 PE (Thermo Scientific, Illinois) and anti-CD62P APC (Biolegend, San Diego). Samples and antibodies were incubated in the dark at room temperature for 15 min. Platelets were identified by forward and side scatter signals, and 15,000 platelet-specific events were analysed by the flow cytometer (BD Accuri C6, BD Biosciences, California) for florescence.

#### Partial thromboplastin time (APTT), prothrombin time (PT) and plasma recalcification time

For the evaluation of APTT and PT, HAp1 and HAp2 and polypropylene beads (negative control) were placed in Eppendorf tubes and were incubated in triplicate with PPP (100 μL; 37 °C, 3 h), at a concentration of 1–10 mg/mL. APTT and PT were determined by using commercial reagents (Diagnostica Stago, Almada, Portugal). Results are reported in seconds.

The plasma re-calcification time was measured to assess the clotting of PPP following activation of prothrombin in the presence of Ca^2+^. HAp1 and HAp2 were placed in 96-well plates (1 and 10 mg/mL/well). PPP (100 μL) was added to each well, followed by the addition of 0.025 M CaCl_2_. The plate was immediately placed in a 96-well plate reader (Biotek Powerwave XS) to monitor the kinetics of the clotting process by measuring the absorbance at 405 nm (every 30 s for 45 min) at 37 °C. Standard 96-well plates (TCP) with PPP and CaCl_2_ served as positive control (formation of the clot), whereas TCP with PPP and without CaCl_2_ was used as negative control (no clot formation). Results are presented as the clotting time to reach half maximal absorbance.

### Endothelial cell response

#### Cell cultures and exposure to HA particles

Human umbilical vein endothelial cells (HUVECs, Lonza) were cultured following a previous procedure [21]. Cultures were established (37 °C, 5% CO_2_/air) in EGM-2 (Lonza). Cells were sub-cultured at 70–80% confluence (0.04% trypsin in 0.25% EDTA solution) and second sub-cultured cells were used in the experiments.

HUVECs were seeded (10^4^ cells/cm^2^) and cultured for 5 days, using the protocol described above. At this stage, the cell layer attained a confluence of 70–80%. HAp1 and HAp2 were added, and cultures were exposed to the particles for 24 h. Cultures performed in standard cell culture plates (in the absence of the particles) were used as control. Cell behaviour was characterized as follows.

#### Dose-dependent cytotoxicity profile

The cytotoxicity profile of HA particles was evaluated by assessing the metabolic activity in a wide concentration range (1 μg/mL to 1 mg/mL), by the MTT assay. This is based in the ability of mitochondrial dehydrogenase, in viable cells, to reduce the MTT (3-(4,5-Dimethylthiazol-2-yl)-2,5,diphenyltetrazolium) to a dark blue formazan product. HUVECs exposed to HA were incubated with MTT (0.5 mg/mL, 3 h, 37 °C). After, the culture medium was removed, the formazan salts were dissolved in dimethylsulphoxide and the Absorbance was determined at λ = 550 nm, on an ELISA reader (Synergy HT, Biotek) and values were normalized by the total cell number, inferred from the quantification of total DNA (Quant-iT PicoGreen dsDNA Assay Kit, Thermo Fisher Scientific). A parallel experiment was run with the particles incubated in culture medium (without cell seeding), using the same protocol as the cell cultures, to compensate for the MTT reduction background levels. Results showed that metabolic activity was affected by HAp1 and HAp2 at levels ≥ 250 μg/mL and 50 μg/mL, respectively. Taking this into account, a detailed evaluation of the endothelial cell response was performed in the cultures exposed to 10 and 50 μg/mL HA particles.

#### Cellular uptake of HA particles

Cells from cultures exposed to HA particles (50 μg/mL) were released (0.04% trypsin in 0.25% EDTA solution, 5 min) and centrifuged (2000 g, 10 min). The cell pellet was fixed (2.5% glutaraldehyde, 2 h), postfixed (2% osmium tetroxide), dehydrated in graded ethanol and embedded in Epon, using standard procedures. Ultra-thin (100 nm) sections mounted in copper grids (300 Mesh) were contrasted with uranyl acetate and lead citrate for TEM analysis (Zeiss EM 10A), at an accelerating voltage 60 kV.

#### F-actin cytoskeleton and CD31 immunolabelling

Cultures exposed to 10 and 50 μg/mL HA particles were fixed (3.7% paraformaldehyde, 15 min), permeabilized (Triton X-100, 0.1%, 5 min) and incubated in bovine serum albumin (BSA/PBS, 1%, 1 h). Cultures were stained for F-actin with Alexa-Fluor-conjugated phalloidin (1:100 in 1% BSA, 1 h; Alexa Fluor^®^ 488 Phalloidin, Molecular Probes) and for cell nuclei with propidium iodide (10 μg/mL, 10 min; Sigma). For CD31 staining, cultures were treated with primary CD31 antibody (1:100 in 1% BSA/PBS, 45 min; PECAM-1 (mouse anti-human antibody, sc-20071; Santa Cruz Biotechnology), then labelled with the secondary antibody (1:1000 in 1% BSA/PBS, 45 min; Alexa Fluor 488 goat anti-mouse IgG1 (ϒ1); Molecular Probes); nuclei were stained with propidium iodide (10 µg/mL, 10 min, Sigma). Samples were observed by Confocal Laser Scanning Microscopy (CLSM; Leica TCP SP2 AOBS microscope). For cell area analysis, sixty cells per specimen were measured using the ImageJ software version 1.50b, National Institute of Health, USA.

#### Apoptosis and Cell cycle analysis

Cultures exposed to 10 and 50 μg/mL HA particles were analysed for the alterations in the apoptotic cell index and cell cycle analysis, using aliquots of 2 × 10^6^ cells. The relative percentage of cells undergoing early and late apoptosis was analysed with TACSTM Annexin V-FITC Apoptosis Detection Kit (R&D Systems), following manufacturer instructions. For cell cycle analysis, cells were fixed (70% ethanol), washed in PBS, centrifuged (500*g*, 7 min) and stained in PBS containing 50 μg/mL PI and 50 μg/mL RNAse (20 min, in the dark at room temperature). Apoptotic cells’ index and cell cycle analysis were performed in an Imaging Flow cytometer ImageStreamX (Amnis Corporation, Seattle, WA) and further evaluated with FlowJo X software (Treestar, Ashland, OR).

#### Gene expression by qPCR

HUVECs exposed to 50 μg/mL HA particles were evaluated for quantitative gene expression. Total RNA was extracted using the Nucleospin RNA II kit (Machery-Nagel), according to the manufacturer’s instructions. The concentration and quality of RNA was determined by NanoDrop (NanoDrop Technologies). cDNA was generated using QuantiTect RT Kit (Qiagen) and qPCR was performed using an iCycler iQ Real-time PCR system (Bio-Rad), with the QuantiTect SYBR green PCR Kit (Qiagen). QuantiTech Primers were: vWF (QT00051975), PECAM-1 (QT00081172), VE-Cadherin (QT00013244) and GAPDH (QT000079247). The relative quantification of each target gene, normalized to GAPDH levels was calculated via the 2^–ΔΔCt^ method.

#### Organization of the HUVECs in tubular-like structures

The ability of endothelial cell cultures to organize a continuous cell layer in a network of tubular-like structures upon the contact with an extracellular matrix was evaluated using the Matrigel assay. HUVECs were exposed to 10 and 50 μg/mL HA particles and, subsequently, the medium was removed and the cultures were covered with a layer of Matrigel matrix (Matrigel Basement Membrane; Sigma). Upon the jellification of the Matrigel matrix (30 min, 37 °C), culture medium was added to the cultures. After 24 h, cultures were stained for F-actin cytoskeleton and nucleus (as described above), and were observed and photographed by CLSM. Tube length and branching points (considered where two tubes grew out of a single tube) data was determined following ImageJ software (National Institutes of Health, Bethesda, MD) analysis, and normalized by values of the control.

#### Statistical analysis

Three independent experiments were performed; in each experiment, five replicas were set up for the quantitative assays and three replicas for the qualitative assays. Results are presented as mean ± standard deviation, and data were analysed by one-way analysis of variance followed by a Bonferroni post hoc test. A p value ≤ 0.05 was considered statistically significant.

## Results

### HA particles

**Fig. 1:**
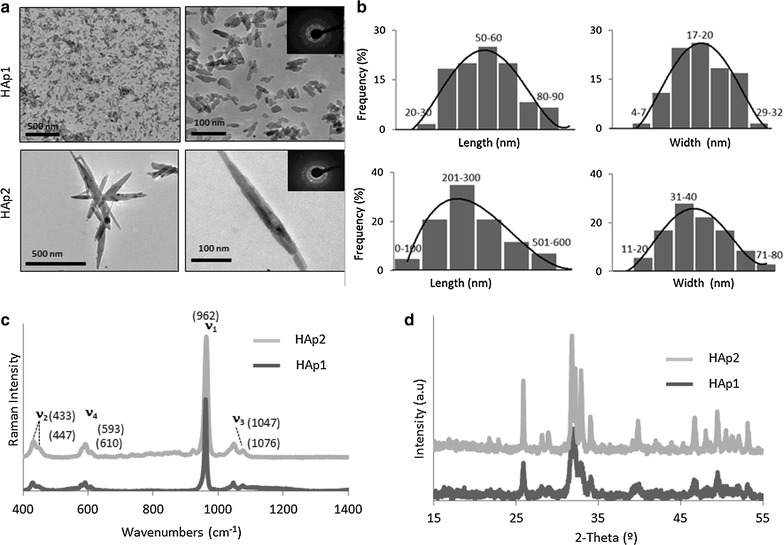
Characterization of HAp1 and HAp2: TEM images (**a**) and the corresponding electron diffraction patterns (insets), particles’ size distribution (**b**), FTIR (**c**) and DRX (**d**) spectra. HAp1 presented rod-like morphology with nanometric width and length dimensions, and low aspect ratio, and HAp2 appeared needle-like with the width in the nanometric range and the length attaining micro-dimensions, exhibiting a high aspect ratio. FTIR spectrum showed the specific bands of pure phase hydroxyapatite and XRD spectrum indicated that hydroxyapatite is the single crystalline phase present in both particles. DRX patterns were in good accordance with the standard data for the pure phase hydroxyapatite

On TEM observation, HA particles had distinguished morphologies, sizes and aspect ratios, Fig. 1a, b. HAp1 presented rod-like morphology, with low aspect ratio (width ~ 15–30 nm, length ~ 40–70 nm). HAp2 exhibited needle-like shape, and high aspect ratio (width ~ 30–60 nm, length ~ 200–500 nm). The SAED patterns of both samples displayed the characteristic diffraction patterns of polycrystals, Fig. 1a, insets. EDX analysis identified only oxygen, calcium and phosphorus elements, which is in agreement with the elemental composition of hydroxyapatite. The specific surface area was 100 ± 5 and 65 ± 6 m^2^/g, respectively for HAp1 and HAp2.

Both particles presented similar Raman spectra, Fig. 1c, with the specific bands of the PO_4_^3−^ groups which are characteristic of pure phase hydroxyapatite. The ν1(PO_4_) band at 962 cm^−1^ was very intense in both spectra which is specific of synthetic hydroxyapatite particles. This vibration mode was associated with the totally symmetric (ν1) P-O-P stretching mode of the free tetrahedral phosphate ion. The other detected phosphate modes at 1076 cm^−1^ (ν3(PO_4_)), 1047 cm^−1^ (ν3(PO_4_)), 593 cm^−1^ (ν4(PO_4_)), 610 cm^−1^ (ν4(PO_4_)), 433 cm^−1^ (ν2(PO_4_)) and 447 cm^−1^ (ν2(PO_4_)) were less expressive, but present. The DRX patterns of both particles, Fig. 1d, were in good agreement with the standard JCPDS data (Nº 09-0432) for the pure phase of hydroxyapatite.

### In vitro blood compatibility

The percentage of haemolysis with HAp1 and HAp2, at 1 and 10 mg/mL, was ~ 0.25%, being only slightly higher than that found in the negative control (polypropylene; ~ 0.21%).

**Fig. 2:**
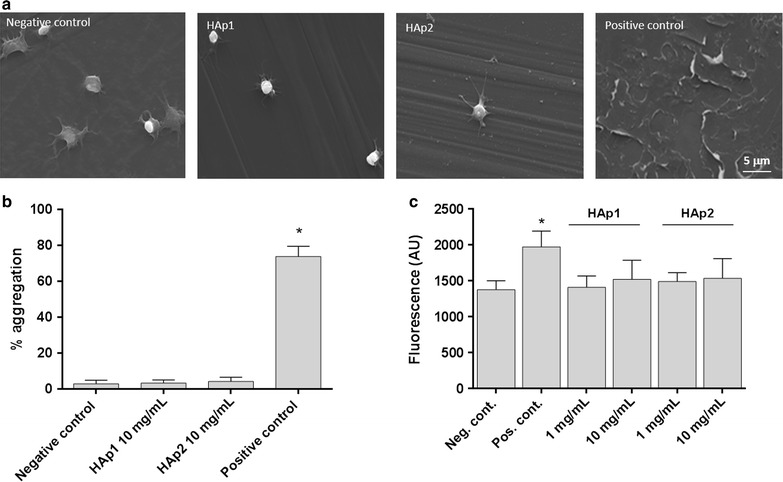
Effect of HAp1 and HAp2 on platelet functionality: SEM images of platelet adhesion and morphology in the presence of HA particles (10 mg/mL) (**a**), light aggregometry (**b**) and expression of CD62P by platelets (1 and 10 mg/mL HA) (**c**)

For the platelet adhesion and aggregation assay, platelet rich plasma was exposed to both HA particles, 10 mg/mL. SEM observation (Fig. 2a) showed that platelets maintained the round shape but formation of some spread pseudopodia was noted. Similar morphology was observed on the polypropylene surface used as a non-thrombogenic control material (negative control). For comparison, morphology of platelets on glass (positive control) is also presented, showing aggregated platelets with a completely flat/spread morphology. PRP incubation with HAp1 or HAp2 particles (10 mg/mL) did not induce platelet aggregation as determined by light aggregometry, revealing values similar to the negative control (Fig. 2b). Further, in another assay, HA particles, 1 and 10 mg/mL, did not significantly affect the expression of CD62P (P-selectin), Fig. 2c.

**Fig. 3:**
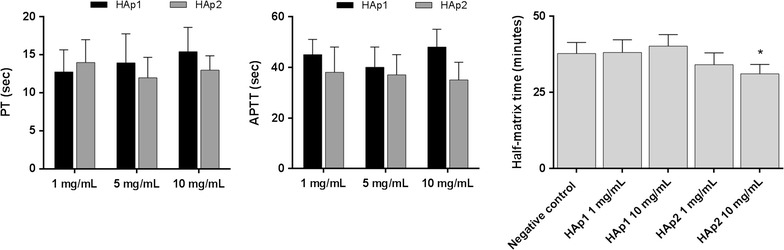
Effect of HAp on coagulation: APTT, PT and Plasma recalcification time (1–10 mg/mL HAp). Both particles had minimal effects on blood compatibility assays, but HAp2 revealed a trend for decreased values at 10 mg/mL. *Significantly different from negative control; p ≤ 0.05

On the coagulation assays, performed in platelet poor plasma, HAp1 and HAp2 (1–10 mg/mL) did not have a major influence on APTT and PT, and also in the plasma recalcification assay (Fig. 3). However, in all assays, a trend for decreased values were noted for HAp2, at 10 mg/mL.

### Endothelial cell response

#### Dose-dependent cytotoxicity

**Fig. 4:**
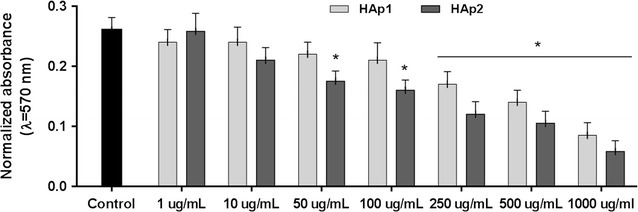
Metabolic activity of human endothelial cells exposed to HA particles, 1–1000 μg/mL. HAp1 and HAp2 decreased cell viability at levels ≥ 250 μg/mL and ≥ 50 μg/mL, respectively. *Significantly different from control (absence of particles); p ≤ 0.05

HUVECs exposed to HA particles, 1–1000 μg/mL, were evaluated for the metabolic activity. HAp1 caused inhibitory effects at levels ≥ 250 μg/mL (~ 30–70%) and HAp2 at levels ≥ 50 μg/mL (~ 15–90%) (Fig. 4). Detailed endothelial cell response was analysed in cultures exposed to 10 and 50 μg/mL HA, as follows.

#### Cellular uptake

**Fig. 5:**
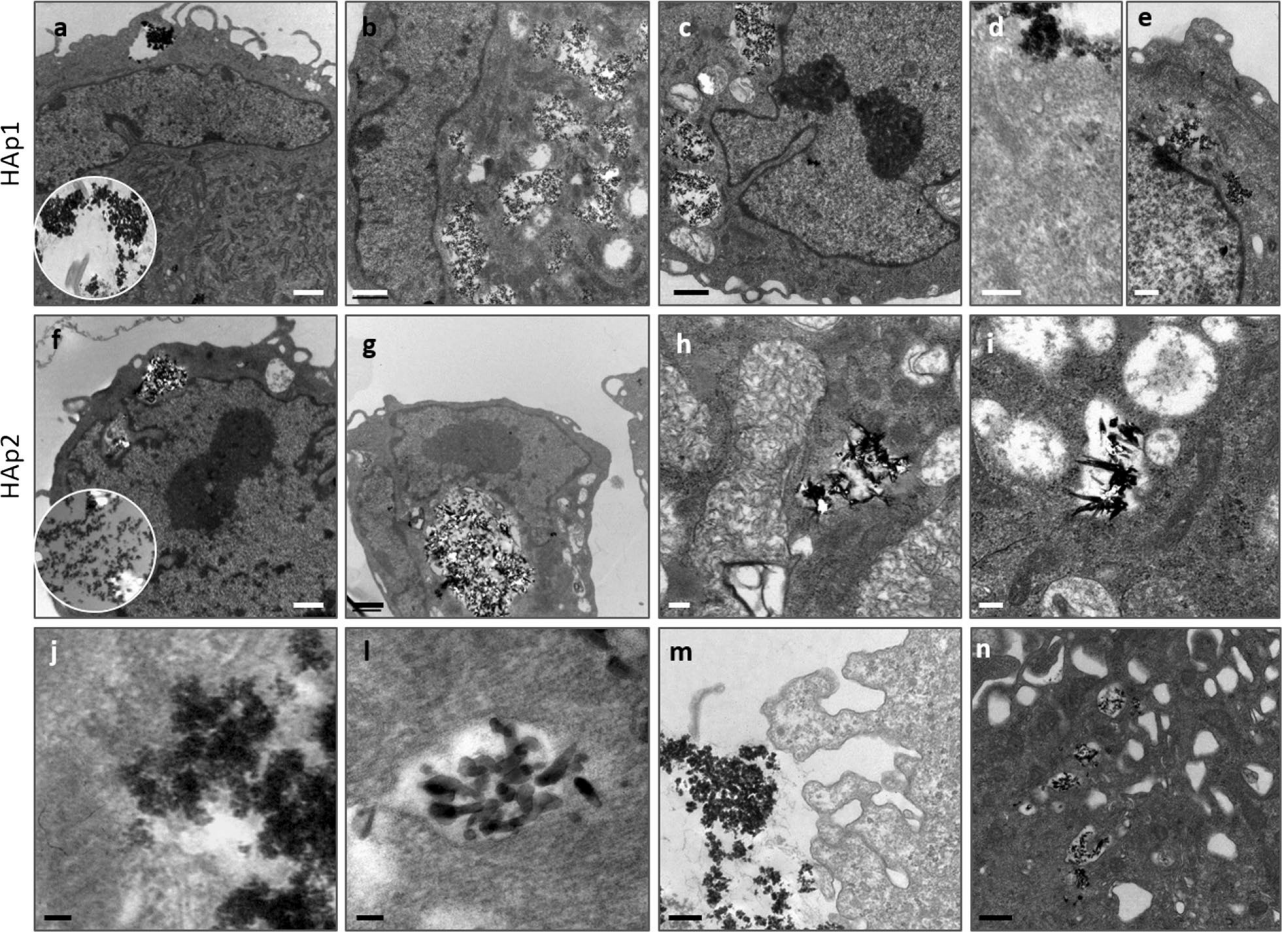
Representative TEM images of human endothelial cells exposed to HA particles (50 μg/mL). The cytoplasm contained a variable number and size of vesicles loaded with the particles (**a**–**c**, HAp1; **f**–**h**, HAp2). High magnification images showed a close interaction of the particles with the cell membrane (**d**, HAp1). Particles did not enter the nucleus (**e**, HAp1), maintained their characteristic size and shape inside the vesicles (**i**, HAp2; **j**, HAp1) and evidence of particle dissolution was attained (**j**, HAp1; **l**, HAp2). In the cell vicinity, particles are seen mostly as aggregates (**m**, HAp1) and close to these structures, cell membrane exhibits a characteristic morphology with the emission of abundant and large pseudopodia extensions (**m**, HAp1) that appear very efficient in entrapment (**n**, HAp2). Insets: extracellular aggregates, HAp1 (**a**) and HAp2 (**f**). Bar: **a**–**c**, **f**, **g** = 1 μm; **e**, **m**, **n** = 500 nm; **d**, **h**; **i** = 200 nm; **j**, **l **= 50 nm

Endothelial cells exposed to 50 μg/mL HA were observed by TEM, Fig. 5. In the cell surroundings, particles were seen mostly as aggregates of variable sizes. HAp1 formed larger and more compact aggregates compared to HAp2. Close to these aggregates, cells showed the profusion of the cell membrane in large pseudopodia (sometimes delimiting vacuoles) that appeared very efficient in entrapping the particles’ aggregates by endocytic mechanisms. After 24 h exposure, the cytoplasm contained variable number and size vesicles loaded with particle aggregates. Again, inside the vesicles, HAp1 aggregates could be identified with distinct morphological characteristics—some aggregates were large and rounded and the particles maintained the rod-shape morphology, whether other, particularly at high magnifications, were found to present a more homogenous structure, supporting HAp dissolution. HAp2 aggregates with distinct morphological features could further be identified—from rounded to irregular appearances sometimes displaying well-defined needle-like particles facing the surroundings, as well as more homogenous structures, a suggestive evidence of particle dissolution—particularly at high magnification images. There is no evidence of the presence of the particles in the nucleus or in other organelles.

#### F-actin cytoskeleton organization

**Fig. 6:**
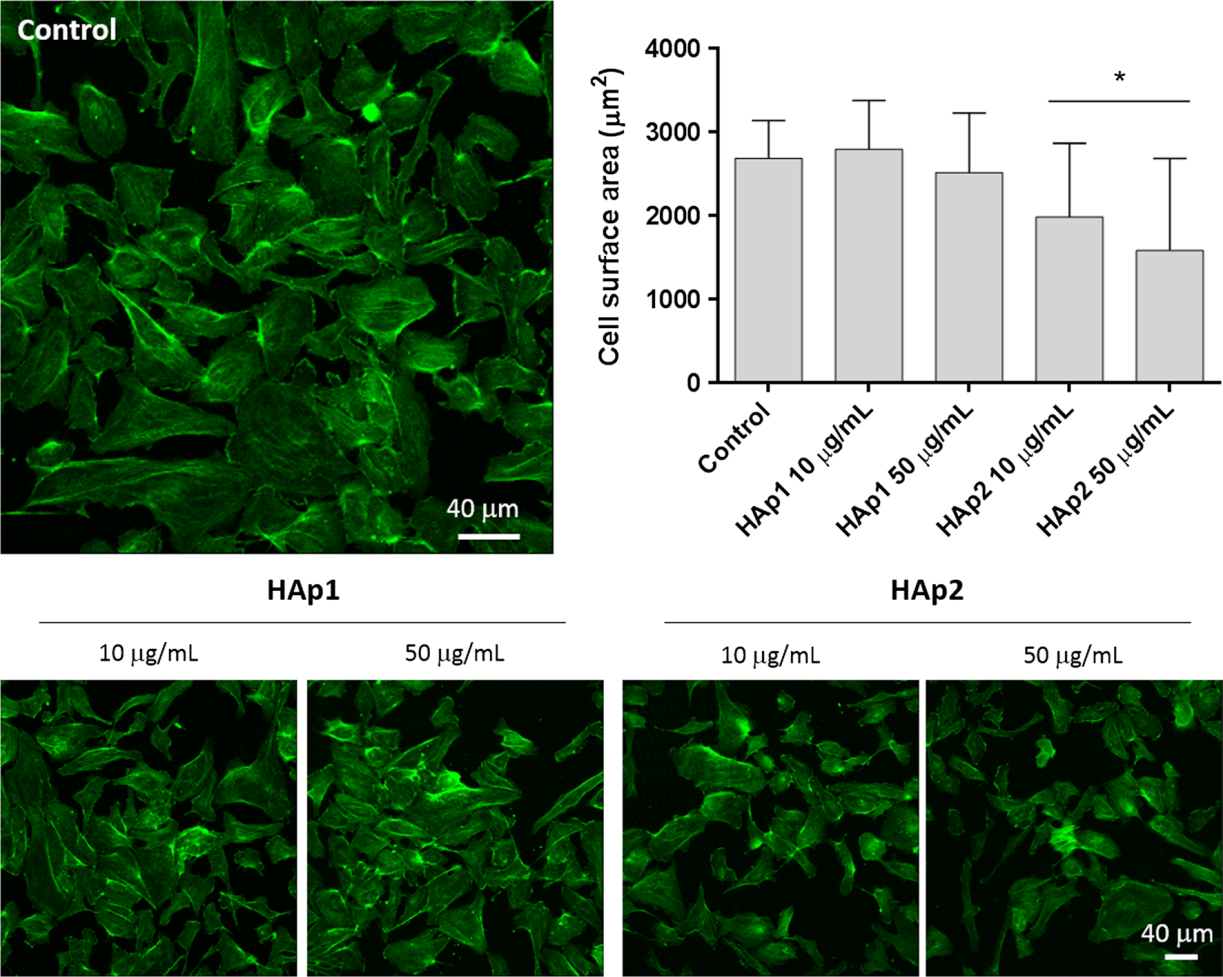
CLSM images of human endothelial cells exposed to HA particles (10 and 50 μg/mL) and stained for F-Actin cytoskeleton. On control cells, the cytoskeleton was organized in a network of well-defined and filamentous stress fibres; HAp1 had little effect in this behaviour, whereas HAp2 caused dose-dependent disruption in the cytoskeleton that was shown by diffuse F-actin organization, less stress fibre formation and F-actin condensation (particularly with 50 μg/mL)

Figure 6 shows CLSM images of endothelial cells exposed to 10 and 50 μg/mL HA and stained for F-actin cytoskeleton. Control cells displayed round-elongated morphology with a high degree of cell spreading. Overall, HAp1 had little effect on this behaviour, whereas HAp2 caused a significant reduction on the cell surface area.

#### Apoptosis and cell-cycle

**Fig. 7:**
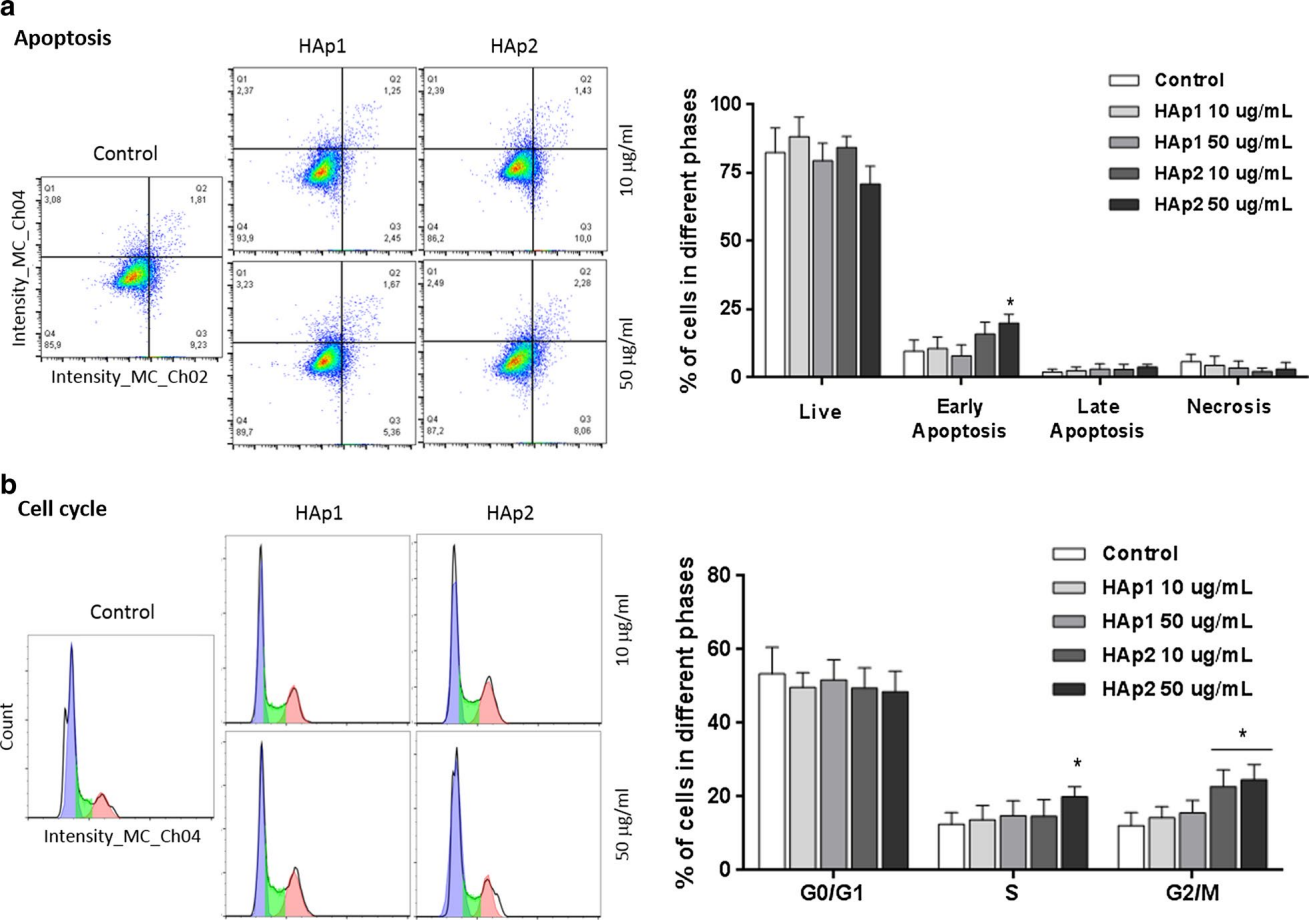
Cell apoptotic index (**a**) and cell cycle analysis (**b**) of human endothelial cells exposed to HA particles (10 and 50 μg/mL)—representative flow cytometry histograms and quantitative data. HAp2 caused a small increase in the % of early apoptotic cells and in the % of cells in the S-phase and G2/M-phase. *Significantly different from control (absence of particles); p ≤ 0.05

Control cultures presented low apoptotic index, Fig. 7a. Exposure to HAp1, 10 and 50 μg/mL, had no noticeable effect, whereas HAp2 (50 μg/mL) increased slightly the  % of early apoptotic cells. On the cell cycle analysis, Fig. 7b, HAp2 caused a small increase in the % of cells in the S-phase and G2/M-phase.

#### Endothelial phenotype

**Fig. 8:**
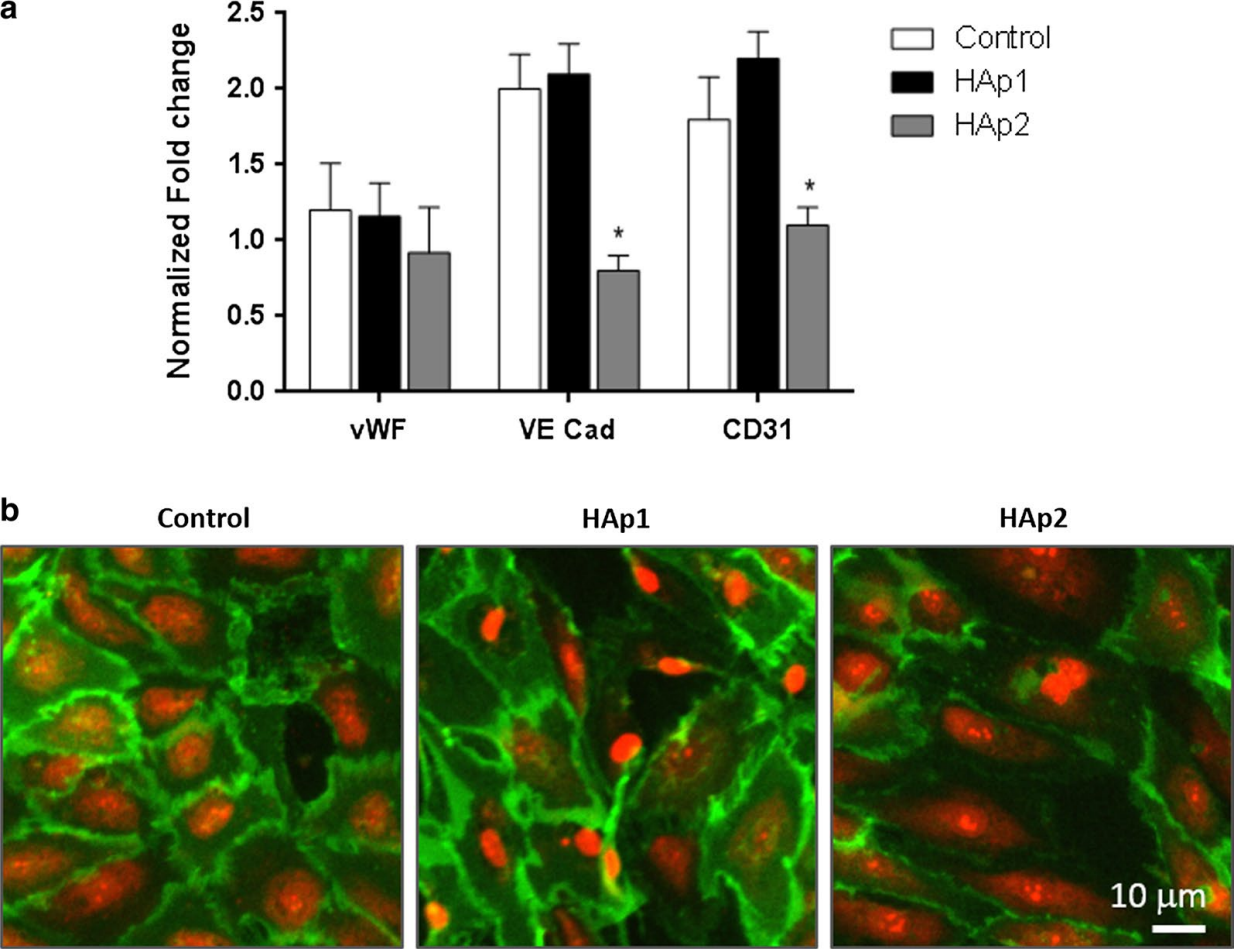
Gene expression of vWF, CE Cad and CD31 by human endothelial cells exposed to HA particles (**a**) and CLSM images of cells stained for CD31 (green) and nucleus (red) (**b**); HA particles: 50 μg/mL. HAp2 decreased the expression of VE Cad and CD31 genes, and affected the immunolabeling of CD31. *Significantly different from control (absence of particles); p ≤ 0.05

Endothelial cells exposed to HA particles, 50 μg/mL, were analysed for the expression of vWF, VE Cad and CD31, Fig. 8a. HAp1 did not affect the expression of these genes, whereas HAp2 caused decreased expression of VE Cad and CD31. Immunolabeling of CD31, Fig. 8b, was consistent with this observation. In control cultures, cells were organized as a continuous cell layer with tight cell-to-cell junctions that stained intensively for CD31.With Hap1, a similar cell organization and CD31 staining was verified while with Hap2, loss of cell layer integrity was observed along with decreased CD31 staining.

**Fig. 9:**
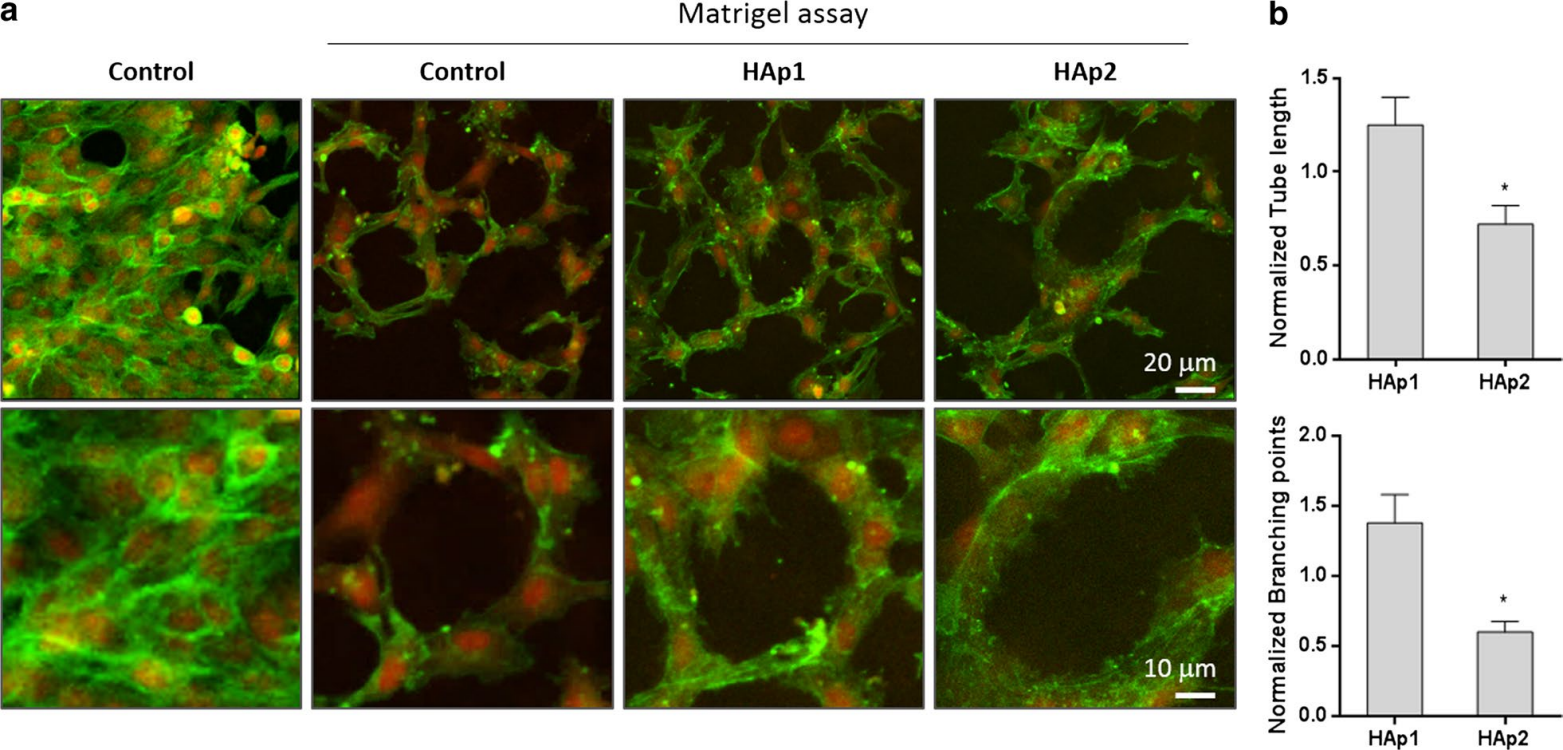
Representative CLSM images of endothelial cells stained for F-actin cytoskeleton (green) and nucleus (red) upon the addition of Matrigel (**a**): control cultures before and after the addition of Matrigel, and cultures exposed to HA particles (50 μg/mL) after the addition of Matrigel; and, quantitative analysis of tube length and branching points following the addition of Matrigel (**b**). Control cells re-organized the continuous cell layer in a tubular-like network upon the contact with Matrigel. HAp1 had a similar behaviour, but HAp2 disrupted significantly this functional feature. *Significantly different from control (absence of particles); p ≤ 0.05

Figure 9a shows CLSM images of endothelial cells stained for F-actin cytoskeleton and nucleus, after contacting with an extracellular matrix (Matrigel). In control cultures, the continuous cell layer re-organized in a well-defined tubular-like network upon contacting with Matrigel. HAp1 allowed the formation of a similar organization but HAp2 caused a disruption in the tubular-like re-organization of the cell layer, reflected by a lower definition of these structures, fewer branch points and shorter tube length (Fig. 9b).

## Discussion

The presence of particulate material in the blood stream may interfere with the homeostatic balance within the vascular compartment [3], which is maintained by the interplay of the endothelium, platelets and the plasma coagulation cascade [6]. This work was set up to evaluate the dose-effects of HA particles on these three components, focusing the relevance of a multifaceted approach to establish the vascular biosafety profile. This was addressed using commercial HA particles, due to their biomedical significance in distinct clinical applications, ranging from bulk implants for mineralized tissues, to particles for biomolecules-delivery or tissue repair. Despite the wide range of established and potential applications, there is a scarcity of data on their vascular compatibility.

On TEM images, HAp1 presented rod-like morphology with nanometric width and length dimensions (< 100 nm) and low aspect ratio. HAp2 appeared needle-like with the width in the nanometric range and the length attaining micro-dimensions (0.2–0.5 μm), thereby exhibiting a high aspect ratio. Accordingly, HAp1 had higher surface area compared to HAp2. Raman spectra, which provide information on the composition and on the surface groups, were similar showing the specific bands characteristic of pure phase hydroxyapatite [22]. The DRX indicated that hydroxyapatite is the single crystalline phase present in both particles.

Blood compatibility of HA particles was evaluated for haemolysis, platelet activation and aggregation, and coagulation, at 1–10 mg/mL.

Exposure to HAp1 and HAp2, up to 10 mg/mL, did not affect erythrocyte integrity, as the percentage of hemolysis, measured by the release of haemoglobin, was in the order of 0.25%. According to ASTM F756-13 [18], these particles may be considered non-haemolytic (haemolysis percentage < 2%). Similar results were reported for needle-shape nanoparticles tested in mice erythrocytes at 10 mg/mL [10] and rod-like nanoparticles assayed in human erythrocytes, up to 4 mg/mL [13].

Additionally, on assays performed in platelet rich plasma (PRP), HAp1 and HAp2 at 10 mg/mL had little effect on the platelets’ functionality [6, 23]. Platelets are very sensitive to changes in the blood microenvironment being activated by a number of different stimuli, as physiological molecules (e.g. thrombin, collagen, immunoglobulins), microorganisms, drugs and particulate compounds [24]. Platelet activation is associated with changes in morphology, development of pseudopodia, activation of cell membrane markers and release of signalling molecules and pro-coagulant compounds, together leading to platelet aggregation [24]. Both particles induced minimal platelet activation, considering the five-point scale relating morphology with increasing degree of activation: rounded, dendritic, spread-dendritic, spread, and fully spread [24]. This was further confirmed by the light aggregometry assay and the verified absence of effect of HA particles at 1 and 10 mg/mL, on the expression of the platelet surface activation marker P-selectin (CD62P), known to promotes platelet aggregation through platelet–fibrin and platelet-platelet binding [23]. These results are in line with previous ones performed in rod-like HA nanoparticles, using similar concentrations [13].

The effect of HA particles was also analysed on the coagulation cascade, a sequence of proteolytic reactions that may be activated within the plasma by surface-mediated events (intrinsic pathway) and also upon vascular/endothelial injury and the contact of blood with the sub-endothelial tissues (extrinsic pathway). Both pathways lead to the formation of thrombin that converts soluble fibrinogen into an insoluble fibrin network that stabilizes the platelet aggregates to form a blood clot (thrombus). In vivo, these proteolytic reactions occur mostly on anionic phospholipid membrane surfaces, from activated platelets or endothelial cells, and require calcium as a cofactor [23]. Platelet poor plasma (PPP) was used to address APTT and PT to screen, respectively, the intrinsic and the extrinsic coagulation pathways. Further, the plasma recalcification time, also giving information on the intrinsic pathway activation, was analysed. Only HAp2 (at 10 μg/mL) caused a slightly decrease in these parameters, suggesting an eventual thrombotic potential. Overall, these results agree with previous studies showing that rod-like HA nanoparticles tested up to 4 mg/mL in human PPP had little interference with these assays [13].

Once in the blood flow, particulate materials interact also with the endothelium, a well-organized monolayer of endothelial cells lining the inner surface of the blood vessels. The effects of HAp1 and HAp2 were analysed in endothelial cell cultures forming a pre-confluent layer of metabolic active cells, in an attempt to better mimic the continuous in vivo endothelium. HAp2 decreased cell viability at significantly lower concentrations (≥ 50 μg/mL) compared to those of HAp1, detectable only at high levels (≥ 250 μg/mL). This is in line with a recent study involving the exposure of HUVECs to HA near-spherical and rod-like nanoparticles and microparticles up to 200 μg/mL [16]. In the present work, a detailed endothelial cell response was performed after exposure to HAp1 and HAp2 at 10 and 50 μg/mL.

On TEM observation, HAp1 and HAp2 appeared in the cell surroundings as particle’ aggregates. In biological fluids (in this case, the culture medium containing foetal bovine serum), the rapid adsorption of a protein layer on the particles’ surface, greatly contributes to the formation of these aggregates [25]. This is a common finding in several cell types interacting with HA particles [26–31]. In the present work, it is worth to note that close to the aggregates, the cell membrane exhibited a typical arrangement, forming a kind of network of large pseudopodia open to the extracellular space in some points, suggesting an activated state towards the aggregates internalization. This behaviour was previously emphasized for the interaction of HA nano and microparticles with human monocyte-derived macrophages [32, 33] and also in endothelial cells, in a monocyte-endothelial cell co-culture model [29]. The degree of agglomeration is known to affect many aspects of particles-cells interactions, greatly determining total uptake [32–35]. The formation of such branched interconnected cell surface arrangements has been proposed as a protective mechanism due to the rapid sequestering of large particles’ agglomerates [33]. In the present work, the HA aggregates were internalized and exclusively located in endocytic vesicles, showing frequently signs of particle dissolution/degradation, as observed before in several cell types [26–32]. Cellular uptake of HA nano- and microparticles by HUVECs exposed to 25 μg/mL was recently addressed [16]. This study suggested that the internalization of these particles by HUVECs occurred mainly by a clathrin- and caveolin-mediated endocytosis, but actin-dependent micropinocytosis also seemed to play a role [16].

The organization of the F-actin filamentous cytoskeleton and cell spreading was affected upon exposure to HAp2, as observed on CLSM. Cytoskeleton provides structural stability and is highly dynamic to cope with cellular internal needs and response to the surrounding environment, thus being essential in functions such as cell division, intracellular transport, signalling pathways and gene expression, as well as in cell motility and mechano-transduction mechanisms associated with differentiation events [36]. Accordingly, adverse effects on F-actin cytoskeleton are early indicators of cytotoxicity [36]. Previously, alterations in this structure were reported on bovine post-capillary venular endothelial cells exposed to 10 μg/mL HA nanocrystals [14] and HUVECs in contact with 25 μg/mL HA nano- and microparticles [16]. HAp2, particularly at 50 μg/mL, also caused a small increase in the percentage of early apoptotic cells and a delay in the cell cycle progression, this suggesting some replicative stress in the cell population proliferative status. A similar tendency was reported in HUVECs exposed to 100 and 200 μg/mL HA nano- and microparticles [16].

Regarding phenotype features, both HAp1 and HAp2 did not affect the expression of vWF gene, coding for a glycoprotein produced by endothelial cells that plays major roles in blood coagulation. HAp2 (50 μg/mL) decreased the expression of VE Cad and CD31 genes, coding for proteins with a key role in endothelial cell-to-cell junction and maintenance of endothelium integrity [37]. In accordance with the gene expression assay, immunolabeling of CD31 was also affected after exposure to HAp2.

In vitro, functional-competent endothelial cells have the ability to re-organize a continuous cell layer in a tubular-like network, upon the contact with an extracellular matrix [38]. This assay would mimic the in vivo capillary formation during angiogenesis involving matrix remodelling and organization of endothelial cells in a 3D network [38]. Also, in vitro, such a process implies the ability of endothelial cells to secrete proteases to invade the gel and re-organize the cell layer to form tubular-like structures. In the present work, this behaviour was clearly observed in control and Hap1 exposed cultures. However, HAp2 disrupted this functional feature, in a concentration-dependent way. The decreased expression of VECad and CD31, seen after exposure to HAp2, might contribute to this effect, given the deterministic role of these molecules on endothelial cell-to-cell interaction [37]. A similar finding was observed in HUVECs exposed to HA nano- and microparticles (50 μg/mL) for 8 h [16].

Summarizing, at 10–50 μg/mL, endothelial cell behaviour was not affected by HAp1 but HAp2 induced dose-dependent deleterious effects. The two particles differ on morphology, size and specific surface area, and aspect ratio, which directly influence surface reactivity and, thus, the particles’ behaviour in biological fluids containing complex ionic and molecular compositions. The prompt protein adsorption to the particles’ surface leads to the formation of protein/particles entities, complex dynamic structures in constant re-organization events to conciliate protein size, charge, relative abundance and affinity to the particles [39, 40]. This dynamic interactive behaviour is driven by the particles’ multiple features that direct surface reactivity [41]. Regarding this, large specific surface area and uniform size/aspect ratio may favour the formation of bigger and more stable particle-biomolecule assemblies, with expectable lower reactivity with surrounding cells and media, as previously advanced in a study addressing several nanoparticles with different physicochemical features [42]. Consistent with this, in the present work, TEM observation showed that, mostly, HAp1 organized into big, dense and rounded aggregates. Comparatively, HAp2 formed smaller, irregularly shaped aggregates that, frequently, exhibited the individual needle-like particles facing the surroundings, thus more prone to direct interactions compared to the rounded HAp1 aggregates. The aggregation profile of HAp2 is likely to be favoured by the particles’ features, namely the high length/width ratio, wide size distribution and needle-shaped morphology, which hinder a homogeneous particles’ fitting. This is in line with previous studies on the shape-dependent effects of HA particles in several cell types showing that needle-shaped particles exhibited higher cytotoxicity compared to rod-like particles [43–45]. The HA particles features/agglomeration profiles would also affect the degradation/dissolution rate occurring in the extracellular environment and inside the intracellular vesicles, leading to an increase in Ca^2+^ levels. Calcium ion is a key modulator of cell behaviour and appropriate extra- and intracellular levels are needed to cellular homeostasis [46]. The smaller and irregularly shaped HAp2 aggregates might be associated to a greater instability and disassembly compared to the more homogeneous HAp1 agglomerates, leading to differences in the dissolution rate, which might also contribute to the higher HAp2 cytotoxicity. This has been suggested for the variation in extracellular and intracellular Ca^2+^ levels, arising from the dissolution of HA particles [1, 47, 48].

Overall, results showed that endothelial cell behaviour was affected by significantly lower HA levels compared to those that did not interfere with routine blood compatibility assays. The high sensitivity of endothelial cells is of upmost relevance, considering the key role of the endothelium in the vascular homeostasis. An injury in the endothelium activates a local coagulation process that occurs in sequential/overlapping stages. The contact of the plasma with subendothelial cells bearing the membrane-bound tissue factor (TF) triggers local platelet adhesion, activation and aggregation, ensuring an on-going coagulation process in the injured area. In the vascular environment, platelets and the TF-bearing cells have a specialized pro-coagulant role, whereas the endothelium is endowed with anticoagulant features having also unique mechanisms to constrain clot formation in the site of injury and prevent intravascular thrombosis [23]. However, in spite of these integrated and regulated processes, the endothelium responds to injury becoming less anti-thrombotic, which is probably an adaptive mechanism that facilitates haemostasis/coagulation at sites of injury [23]. The great sensitivity of endothelial cells highlights its role in the vascular system dynamics. This is clearly in line with the relevance of the endothelium integrity in maintaining a vascular healthy state [37, 49] and also, the significance of endothelium injury as a major determinant in the genesis and/or progression of a variety of pathological conditions [28, 49–51].

It should be further stressed that, in vivo, the effects of particulate entities in the vascular homeostasis are greatly modulated by their blood clearance rate, a process that is strongly influenced by the vascular components/particles interactions [52, 53]. As any in vitro study, the results reported in this work should be interpreted cautiously. Nevertheless, they suggest that the routine blood platelets and coagulation assays might give partial/limited information, as harmful effects to the endothelium, occurring at significantly lower levels, may compromise vascular homeostasis.

## Conclusion

Vascular homeostasis is a dynamic integrated process involving the endothelium, the platelets and the coagulation system. Accordingly, this work addressed the effects of HA particles, with distinct characteristics regarding size, morphology and aspect ratio, in blood compatibility and human endothelial cells’ functionality. Nanosized rod-like HAp1 and needle-like HAp2 showed similar blood compatibility, but HAp2 exhibited higher toxicity on endothelial cells. However, as a whole, the most relevant observation is the great discrepancy in the HA levels that interfere with the routine blood compatibility assays, focusing haemolysis, platelets and coagulation, and the endothelial cell behaviour, assessed for viability, apoptosis, cell cycle, gene expression and phenotypic expression. Further, this difference was dependent on the particles size, morphology and aspect ratio. The high sensitivity of endothelial cells highlights its determinant role in the dynamics of the vascular system and emphasizes the need of a complementary approach to establish the vascular compatibility of particulate entities.

## Abbreviations

HA: hydroxyapatite
HAp1: rod-like hydroxyapatite particles with nano dimensions and low aspect ratio
HAp1: needle-shape hydroxyapatite particles with wider size and aspect ratio
vWF: von Willebrand factor
VECad: vascular endothelial cadherin
XRD: X-ray diffraction
GIXRD: Grazing incidence X-ray diffraction
TEM: transmission electron microscopy
SAED: selective area electron diffraction
ISTH: International Society on Haemostasis and Thrombosis
BCSH: British Committee for Standards in Haematology
PRP: platelet-rich plasma
PPP: platelet-poor plasma
EDTA: ethylenediaminetetraacetic acid
PBS: phosphate-buffered saline
UV: ultraviolet
APTT: partial thromboplastin time
PT: prothrombin time
HUVECs: human umbilical vein endothelial cells
MTT: 3-(4,5-dimethylthiazol-2-yl)-2,5-diphenyltetrazolium bromide
ELISA: enzyme-linked immunosorbent assay
DNA: deoxyribonucleic acid
RNA: ribonucleic acid
BSA: bovine serum albumin
PECAM-1: platelet endothelial cell adhesion molecule
CLSM: confocal laser scanning microscopy
